# MetaCerberus: distributed highly parallelized HMM-based processing for robust functional annotation across the tree of life

**DOI:** 10.1093/bioinformatics/btae119

**Published:** 2024-02-29

**Authors:** Jose L Figueroa III, Eliza Dhungel, Madeline Bellanger, Cory R Brouwer, Richard Allen White III

**Affiliations:** North Carolina Research Campus (NCRC), Department of Bioinformatics and Genomics, The University of North Carolina at Charlotte, Kannapolis, NC 28081, United States; Computational Intelligence to Predict Health and Environmental Risks (CIPHER) Research Center, Department of Bioinformatics and Genomics, The University of North Carolina at Charlotte, Charlotte, NC 28223, United States; North Carolina Research Campus (NCRC), Department of Bioinformatics and Genomics, The University of North Carolina at Charlotte, Kannapolis, NC 28081, United States; North Carolina Research Campus (NCRC), Department of Bioinformatics and Genomics, The University of North Carolina at Charlotte, Kannapolis, NC 28081, United States; Computational Intelligence to Predict Health and Environmental Risks (CIPHER) Research Center, Department of Bioinformatics and Genomics, The University of North Carolina at Charlotte, Charlotte, NC 28223, United States; North Carolina Research Campus (NCRC), Department of Bioinformatics and Genomics, The University of North Carolina at Charlotte, Kannapolis, NC 28081, United States; North Carolina Research Campus (NCRC), Department of Bioinformatics and Genomics, The University of North Carolina at Charlotte, Kannapolis, NC 28081, United States; Computational Intelligence to Predict Health and Environmental Risks (CIPHER) Research Center, Department of Bioinformatics and Genomics, The University of North Carolina at Charlotte, Charlotte, NC 28223, United States

## Abstract

**Motivation:**

MetaCerberus is a massively parallel, fast, low memory, scalable annotation tool for inference gene function across genomes to metacommunities. MetaCerberus provides an elusive HMM/HMMER-based tool at a rapid scale with low memory. It offers scalable gene elucidation to major public databases, including KEGG (KO), COGs, CAZy, FOAM, and specific databases for viruses, including VOGs and PHROGs, from single genomes to metacommunities.

**Results:**

MetaCerberus is 1.3× as fast on a single node than eggNOG-mapper v2 on 5× less memory using an exclusively HMM/HMMER mode. In a direct comparison, MetaCerberus provides better annotation of viruses, phages, and archaeal viruses than DRAM, Prokka, or InterProScan. MetaCerberus annotates more KOs across domains when compared to DRAM, with a 186× smaller database, and with 63× less memory. MetaCerberus is fully integrated for automatic analysis of statistics and pathways using differential statistic tools (i.e. DESeq2 and edgeR), pathway enrichment (GAGE R), and pathview R. MetaCerberus provides a novel tool for unlocking the biosphere across the tree of life at scale.

**Availability and implementation:**

MetaCerberus is written in Python and distributed under a BSD-3 license. The source code of MetaCerberus is freely available at https://github.com/raw-lab/metacerberus compatible with Python 3 and works on both Mac OS X and Linux. MetaCerberus can also be easily installed using bioconda: mamba create -n metacerberus -c bioconda -c conda-forge metacerberus.

## 1 Introduction

Annotation is a fundamental step in functional gene inference, which is required by many disciplines in biology. Massively parallel sequencing (MPS) has reached the terabyte scale with Illumina NovaSeq X producing 16 Tb per run and Oxford nanopore promethION 7 Tb per run (https://www.illumina.com/systems/sequencing-platforms/novaseq-x-plus.html, https://nanoporetech.com/about-us/news/highest-throughput-yet-promethion-breaks-7-terabase-mark). Due to this increase in MPS, the number of reference microbial genomes and metagenomes has increased by orders of magnitude. Genome Taxonomy Database (GTDB) now includes 402 709 (08-RS214, 28 April 2023) genomes, and the Short Read Archive (SRA) has >4.5 million listed biosample metagenomes (https://gtdb.ecogenomic.org/, Parks *et al.* 2022, https://www.ncbi.nlm.nih.gov/sra/?term=metagenomes). Cellular metagenome-assembled genomes (MAGs) and their viral counterpart vMAGs (viral MAGs) have also rapidly populated public databases through reconstruction from shotgun metagenomics (Bowers *et al.* 2017, Roux *et al.* 2019, Kieft *et al.* 2022). Functional gene annotation is required for metabolic reconstruction, functional, and structural gene differential analysis, inference of pathway regulation, presence/absence of toxin genes (e.g. botulinum toxin A), drug discovery, novel gene cluster discovery (e.g. antibiotic discovery), and viral detection (Kim *et al.* 2010, Machado *et al.* 2018, Santana-Pereira *et al.* 2020, de Nies *et al.* 2021, White III 2021, Zimmermann *et al.* 2021, Zhou *et al.* 2022, Camargo *et al.* 2023a,b). Due to this terabyte scale, the annotation step is often the most prolonged, requiring more CPU time, memory (i.e. RAM), and more resources to finish before obtaining biological insight. Reference databases have also been expanding, which will soon reach the terabyte scale, taking days to download and format, requiring more disk space alongside the data for analysis. Thus scalable, highly parallel, low memory, and fast annotation tools are critical to the future of 'omics analysis.

Functional annotation requires two main steps: gene calling followed by gene assignment to external reference databases using homology or ontology-based approaches. Gene calling finds protein-coding open reading frames (pORFs) alongside ribosomal RNAs, transfer RNAs, and other RNAs. Various tools exist for pORF calling, including Prodigal (or Pyrodigal), FragGeneScanRs, GetOrf, and GeneMark (Besemer and Borodovsky 2005, Hyatt *et al.* 2010, Larralde 2022, Van der Jeugt *et al.* 2022, https://emboss.sourceforge.net/apps/cvs/emboss/apps/getorf.html). Gene assignment of ports to external databases often uses homology-based tools such as BLAST (Camacho *et al.* 2009), MMseq2 (Steinegger and Söding 2017), and/or DIAMOND (Buchfink *et al.* 2015) against databases such as RefSeq (NCBI Reference Sequence Database) (O'Leary *et al.* 2016), UniProt (Universal Protein Resource) (UniProt Consortium 2023), or KEGG (Kyoto Encyclopedia of Genes and Genomes) (Kanehisa *et al.* 2017). Multiple approaches have been applied to matching pORFs, including homology and ontology-based methods. A variety of tools exist that assign pORFs amongst genomes to metagenomes, including Prokka, DRAM (Distilled and Refined Annotation of Metabolism), InterProScan, eggNOG-mapper, and MicrobeAnnotator (Jones *et al.* 2014, Seemann 2014, Shaffer *et al.* 2020, Cantalapiedra *et al.* 2021, Ruiz-Perez *et al.* 2021). Ontology-based approaches are generally superior to homology-based methods (Eddy 2011). InterProScan and eggNOG-mapper use Hidden Markov Models (HMMs) based ontology approaches via HMMER (Jones *et al.* 2014, Cantalapiedra *et al.* 2021) using either KEGG (Kanehisa *et al.* 2017), eggNOG (evolutionary genealogy of genes: Nonsupervised Orthologous Groups) (Hernández-Plaza *et al.* 2023), InterPro (INTEgrative PROtein signature database) (Paysan-Lafosse *et al.* 2023), or Pfam (protein family) databases (Mistry *et al.* 2021). HMMs provide greater sensitivity to elucidate and discover relationships between query and database based on ontology and protein domain-centric (Steinegger *et al.* 2019, Cantalapiedra *et al.* 2021).

Viruses and the candidate phyla radiation (CPRs) have remained challenging to functionally annotate due to limited homology within public databases and the divergent nature of their proteins (Fremin *et al.* 2022, Jaffe *et al.* 2020). DRAM has a specific version (i.e. DRAM-v) to annotate viruses, including the detection of viral auxiliary metabolic genes (vAMGs) (Shaffer *et al.* 2020). Pharokka is a phage specific annotation tool that supports annotation of phage and virome data (Bouras *et al.* 2023). InterProScan, MicrobeAnnotator, and DRAM have attempted to close the gap in CPR functional annotation, with InterProScan annotating the most at ∼75% (Ruiz-Perez *et al.* 2021). While no specific annotation tool or gene database exists for CPR, they are found amongst GTDB and other public repositories (Parks *et al.* 2022). Various databases such as VOGs (Virus Orthologous Groups), pVOGs (Prokaryotic Virus Orthologous Groups), IMG/VR (Integrated Microbial Genome/Virus), INPHARED (INfrastructure for a PHAge REference Database), and PHROGs (Prokaryotic Virus Remote Homologous Groups database) have been introduced to improve annotation viruses from isolates and vMAGs (Cook *et al.* 2021, https://vogdb.org/, Grazziotin *et al.* 2017, Terzian *et al.* 2021, Camargo *et al.* 2023a,b). Still, CPR and viruses remain a significant challenge for functional annotation.

While many tools and databases are present for functional annotation from genomes to metagenomes, gaps remain, including scalability, resource utilization (e.g. memory use), and tools that provide annotation to those with limited functional annotations (e.g. viruses). We present MetaCerberus, a generalizable ontology-based HMM tool that provides scalable, highly parallel, low memory, and fast annotation for genomes to metacommunities across the tree of life.

## 2 Materials and methods

### 2.1 Implementation

#### 2.1.1 Framework and coding base

MetaCerberus is written entirely in Python (version 3) as a wrapper for various other tools described below. Similar to our other software MerCat2 for massively parallel processing (MPP), it utilizes a byte chunking algorithm 1 (‘Chunker’) to split files for MPP for further utilization in RAY, a massive open-source parallel computing framework to scale Python applications and workflows (Figueroa III *et al*. 2022). MetaCerberus can also be single or across nodes with scalable parallelization with RAY. To avoid large RAM consumption, we implemented the greedy algorithm for tab-separated merging and incremental PCA plot limiter from MerCat2 (Figueroa III *et al*. 2022). MetaCerberus utilizes HMM/HMMER exclusively without homology-based tools (e.g. BLAST).

#### 2.1.2 Databases for MetaCerberus

MetaCerberus utilizes KOfams (KEGG protein families) to obtain KEGG KOs (KEGG Ontology) (version 11-Jul-2023, https://www.genome.jp/ftp/db/kofam/), FOAM (Functional Ontology Assignments for Metagenomes), COG (Clusters of Orthologous Genes) (version 2020, https://ftp.ncbi.nih.gov/pub/COG/COG2020/data/), dbCAN (DataBase for automated Carbohydrate-active enzyme ANnotation) for CAZy (Carbohydrate-Active enZYmes Database) ontology for functional gene annotations (version 11, https://bcb.unl.edu/dbCAN2/download/) (Lombard *et al.* 2014, Yin *et al.* 2012, Prestat *et al.* 2014, Aramaki *et al.* 2020, Galperin *et al.* 2021). For viral annotation, MetaCerberus utilizes VOGs (version 219, https://vogdb.org/download), pVOGs (version Sep2016, https://ftp.ncbi.nlm.nih.gov/pub/kristensen/pVOGs/downloads.html#), and PHROGs (version 4, https://phrogs.lmge.uca.fr/) databases (Grazziotin *et al.* 2017, Terzian *et al.* 2021, https://vogdb.org/). FOAM ontology is obtained from KOfam KOs and then computed via a look-up table to avoid redundancy within the current FOAM database version 1. Similar to FOAM, the dbCAN database is used to obtain CAZy ontology via a look-up table. COGs and PHROGs were converted into protein family-specific HMMs (e.g. COG1 -> COG1.hmm) using MAFFT (version 7.273-woe) via local alignments with maximum iterations of 1000 (Katoh and Standley 2013). We compared databases of seven other tools to MetaCerberus, including DRAM, Prokka, InterProScan, MicrobeAnnotator, Bakta, Pharokka, and eggNOG-mapper (Table 1). Bakta, Pharokka, DRAM, Prokka, InterProScan, MicrobeAnnotator, and eggNOG-mapper do not currently support the FOAM and pVOG databases; these are unique to MetaCerberus (Table 1). All tools compared in this study obtain the enzyme commission numbers (EC) numbers, except Pharokka (Table 1). Bakta does not support CAZy (dbCAN), FOAM, VOG, and pVOG (Table 1). Pharokka does not support a majority of the comparison databases but the viral database PHROG, it is missing the main functional databases that MetaCerberus and DRAM-v has which include KEGG (KO), VOG, and CAZy (Table 1).

**Table 1.** Comparing tools based on databases provided.^a^

	EC	KEGG	CAZy	COG	FOAM	VOG	pVOG	PHROG	pfam	EggNOG	InterPro
MetaCerberus	X	X	X	X	X	X	X	X			
DRAM	X	X	X			X			X		
Prokka	X								X		
InterProScan	X								X		X
MicrobeAnnotator	X	X									X
EggNOG-Mapper	X	X	X	X					X	X	
Pharokka								X			

a This includes versions of other databases present within the various tools compared.

X represent Present.

#### 2.1.3 Modes for running MetaCerberus

**Figure 1.**
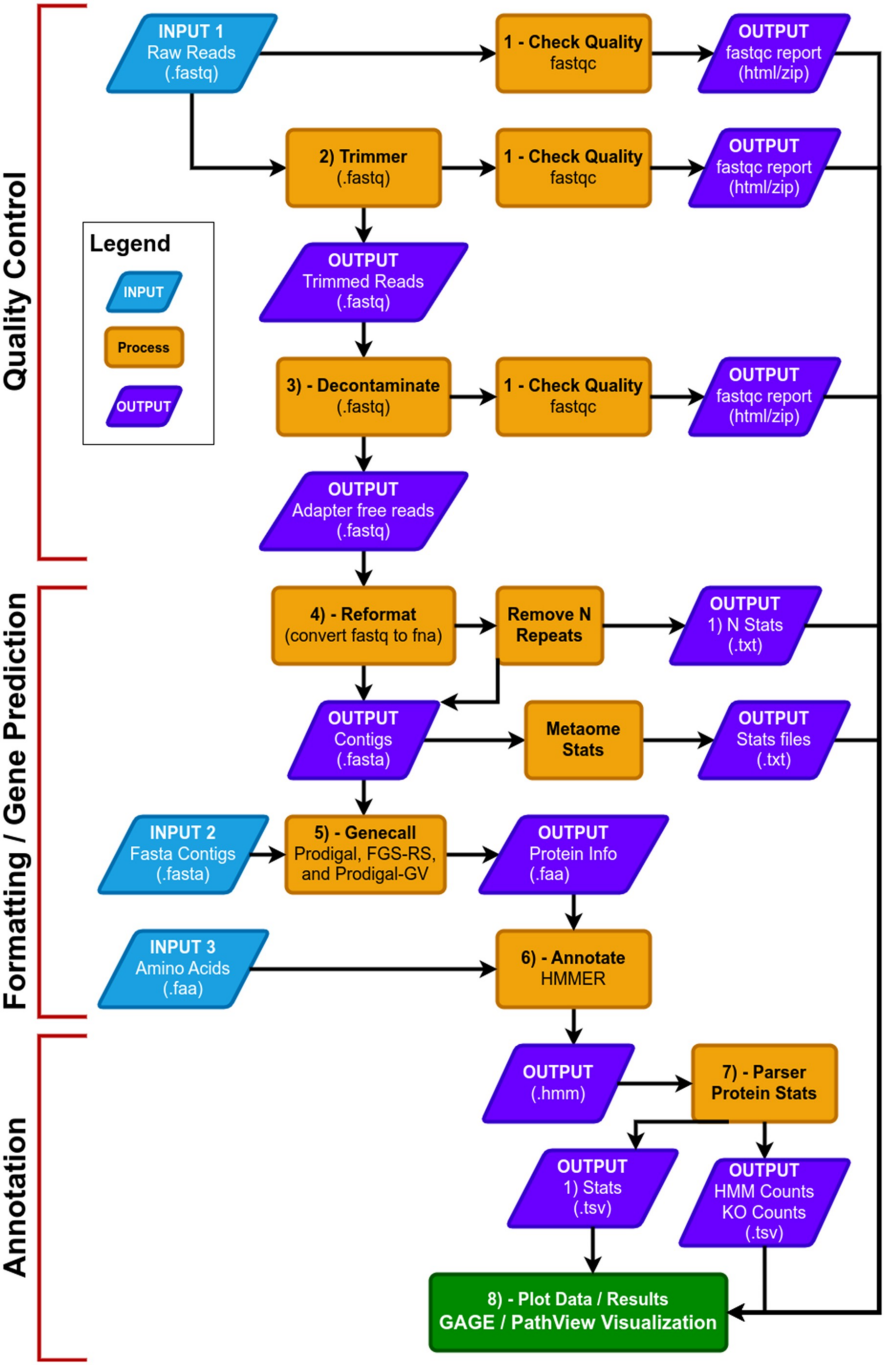
Flowgraph of the MetaCerberus pipeline

MetaCerberus has three basic modes: quality control (QC) for raw reads, formatting/gene prediction, and annotation (Fig. 1). MetaCerberus can use three different input files: (i) raw read data from any sequencing platform (Illumina, PacBio, or Oxford Nanopore), (ii) assembled contigs, as MAGs, vMAGs, isolate genomes, or a collection of contigs, (iii) amino acid fasta (.faa), previously called pORFs (Fig. 1). We offer customization, including running all databases together, individually or specifying select databases. For example, if a user wants to run prokaryotic or eukaryotic-specific KOfams, or an individual database alone such as dbCAN, both are easily customized within MetaCerberus. In future versions, we will provide viral and phage-specific KO modules to run individually. In QC mode, raw reads are quality controlled via fastqc (version v0.12.1) prior and post trim (https://github.com/s-andrews/FastQC). Raw reads are then trimmed via data type; if the data is Illumina or PacBio, fastp (version 0.23.4) is called, otherwise it assumes the data is Oxford Nanopore then Porechop (version v0.2.4) is utilized (Chen *et al.* 2018, https://github.com/rrwick/Porechop, Fig. 1). If Illumina reads are utilized, an optional bbmap (version 39.01) step to remove the phiX174 genome is available. Phage phiX174 is a common contaminant within the Illumina platform as their library spike-in control (Mukherjee *et al.* 2015, Moustafa *et al.* 2017, https://sourceforge.net/projects/bbmap/). We highly recommend this removal if viral analysis is conducted, as it would provide false positives to ssDNA microviruses within a sample. We include a—skip_decon option to skip the filtration of phiX174, which may remove common k-mers that are shared in ssDNA phages.

In the formatting and gene prediction stage, contigs and genomes are checked for N repeats. These N repeats are removed by default. We impute contig/genome statistics (e.g. N50, N90, max contig) via our custom module Metaome Stats (https://github.com/raw-lab/metaome_stats). Contigs can be converted to pORFs using Prodigal, FragGeneScanRs, and Prodigal-gv (Hyatt *et al.* 2010, Van der Jeugt *et al.* 2022, Camargo *et al.* 2023a,b) as specified by user preference (Fig. 1). Scaffold annotation is not recommended due to N's providing ambiguous annotation. Both Prodigal and FragGeneScanRs can be used via our—super option, and we recommend using FragGeneScanRs for samples rich in eukaryotes. FragGeneScanRs found more ORFs and KOs than Prodigal for a stimulated eukaryote rich metagenome (Supplementary Fig. S1, Supplementary Table S1). HMMER searches against the above databases via user specified bitscore and e-values or our minimum defaults (i.e. bitscore = 25, e-value = 1 × 10^−9^).

There are six general rules followed by MetaCerberus for functional annotation (Supplementary Fig. S2). Rule 1 is for finding high quality matches across databases. It is a score pre-filtering module for pORFs thresholds: which states that each pORF match to an HMM is recorded by default or a user-selected cut-off (i.e. e-value/bit scores) per database independently, or across all default databases (e.g. finding best hit), or per user specification of the selected database. Rule 2 is to avoid missing genes encoding proteins with dual domains that are not overlapping. It is imputed for nonoverlapping dual domain module pORF threshold: if two HMM hits are nonoverlapping from the same database, both are counted as long as they are within the default or user selected score (i.e. e-value/bit scores). Rule 3 is to ensure overlapping dual domains are not missed. This is the dual independent overlapping domain module for convergent binary domain pORFs. If two domains within a pORF are overlapping <10 amino acids (e.g. COG1 and COG4) then both domains are counted and reported due to the dual domain issue within a single pORF. If a function hits multiple pathways within an accession, both are counted, in pathway roll-up, as many proteins function in multiple pathways. Rule 4 is the equal match counter to avoid missing high quality matches within the same protein. This is an independent accession module for a single pORF: if both hits within the same database have equal values for both e-value and bit score but are different accessions from the same database (e.g. KO1 and KO3) then both are reported. Rule 5 is the ‘winner take all’ match rule for providing the best match. It is computed as the winner takes all module for overlapping pORFs: if two HMM hits are overlapping (>10 amino acids) from the same database the lowest resulting e-value and highest bit score wins. Rule 6 is to avoid partial or fractional hits being counted. This ensures that only whole discrete integer counting (e.g. 0, 1, 2 to *n*) are computed and that partial or fractional counting is excluded.

#### 2.1.4 Statistics and visualization

MetaCerberus, as previously mentioned, provides genome and contig statistics via MetaOme stats; it also offers seamless integration into automatic differential statistics, visualizations, pathway enrichment, and pathway integration viewing. DESeq2 and edgeR negative binomial distribution differential statistic tools in R are available to users by selection (default is DESeq2) (Robinson *et al.* 2010, Love *et al.* 2014). The outputs from DESeq2, edgeR, or both are automatically enriched for pathway analysis in GAGE (Generally Applicable Gene-set Enrichment for Pathway Analysis) (Luo *et al.* 2009). GAGE outputs are loaded into path view R to visualize differential pathways across user-specified experiments (Luo and Brouwer 2013). These outputs from MetaCerberus include differential KEGG heat maps from Pathview, volcano plots, and heat maps (Luo and Brouwer 2013) (Supplementary Fig. S3). Each genome, contig, and/or read dataset is provided a sample dashboard with a number of pORF called, MetaOme stats (i.e. genome statistics, N50, N90, etc., for genomes/contigs only), PCA with sample sets of >3, and the number of annotated hits (Supplementary Fig. S4).

#### 2.1.5 Across tool comparisons

Tools compared across MetaCerberus (version 1.1) include DRAM (version 1.4.6), InterProScan (version 5.60–92.0), and Prokka (version 1.1). All comparisons were completed on a Dual 8-Core Intel Xeon E5-2667 CPU @ 3.2 GHz (16 cores) using 128 GB RAM. All genomes used in our study are available at https://osf.io/3uz2j/. For further testing of MetaCerberus, we used five distinct genospecies, *Rhizobium leguminosarum*, against five distinct *Exiguobacterium* spp. available at https://github.com/raw-lab/MetaCerberus/tree/main/data/rhizobium_test (Supplementary Table S2).

Viruses from permafrost that were used in the DRAM paper (https://www.ncbi.nlm.nih.gov/nuccore/QGNH00000000) were compared directly to MetaCerberus, Pharokka, and DRAM-v (Shaffer *et al.* 2020, Bouras *et al.* 2023). For statistical comparisons, we computed normality tests (i.e. Shapiro-Wilkes Test), if normal we performed T-tests, and if nonnormal we used nonparametric Wilcoxon rank sum test (i.e. Mann-Whitney U test) in R.

## 3 Results

### 3.1 Database size and download time

Database size, formatting, and downloading are required steps in functional annotation. Substantial databases limit disc storage for data, cost disc space for storage over time, and define the computers that can be used for analysis. We compared download times for various databases required by each tool. These downloads are estimated times based on ∼125 MB/s with 20% overhead to our server. MetaCerberus database size is 3.8 GB, with a download time of ∼4 min, and database format time is zero because they are pre-formatted already for the user (Table 2). DRAM database download requires 710 GB of disc space and requires ∼3 days to download completely (Table 2). According to the DRAM readme, KEGG Genes and UniRef90 need ∼512 GB of disc space and ∼512 GB of RAM for the complete database (26, https://github.com/WrightonLabCSU/DRAM). This database size difference is due to KEGG Genes and UniRef90 updates since their original release in 2020. DRAM can run with more processors within a single node but is not set up for multi-node like MetaCerberus. The InterProScan database is 14 GB, which took ∼2.45 h to install (Table 2). Prokka had the smallest database at 636 MB and was the fastest installed at ∼3 ½ minutes (Table 2). MicrobeAnnotator requires at least ∼237 GB for its full version and ∼0.65 GB for its light version (Table 2).

**Table 2.** Database size, download, and formatting time across tools.^a^

Tool	Time	Disk	Version
DRAM	∼ 3 days	∼710GB	v1.4.6
InterProScan	∼ 2:45:59.23	14GB	v5.60–92.0
Metacerberus	∼ 0:04:14.29	3.8GB	v1.1
PROKKA	∼ 0:03:28.68	607M	v1.14.6
EggNOG-Mapper	∼14:33:31.74	31GB	V2.1.8
MicrobeAnnotator	>3 days	∼237GB	v2.0.5

a These estimates of download speed are based on ∼125 MB/s with 20% overhead to our server.

### 3.2 Computational resource comparison

**Figure 2.**
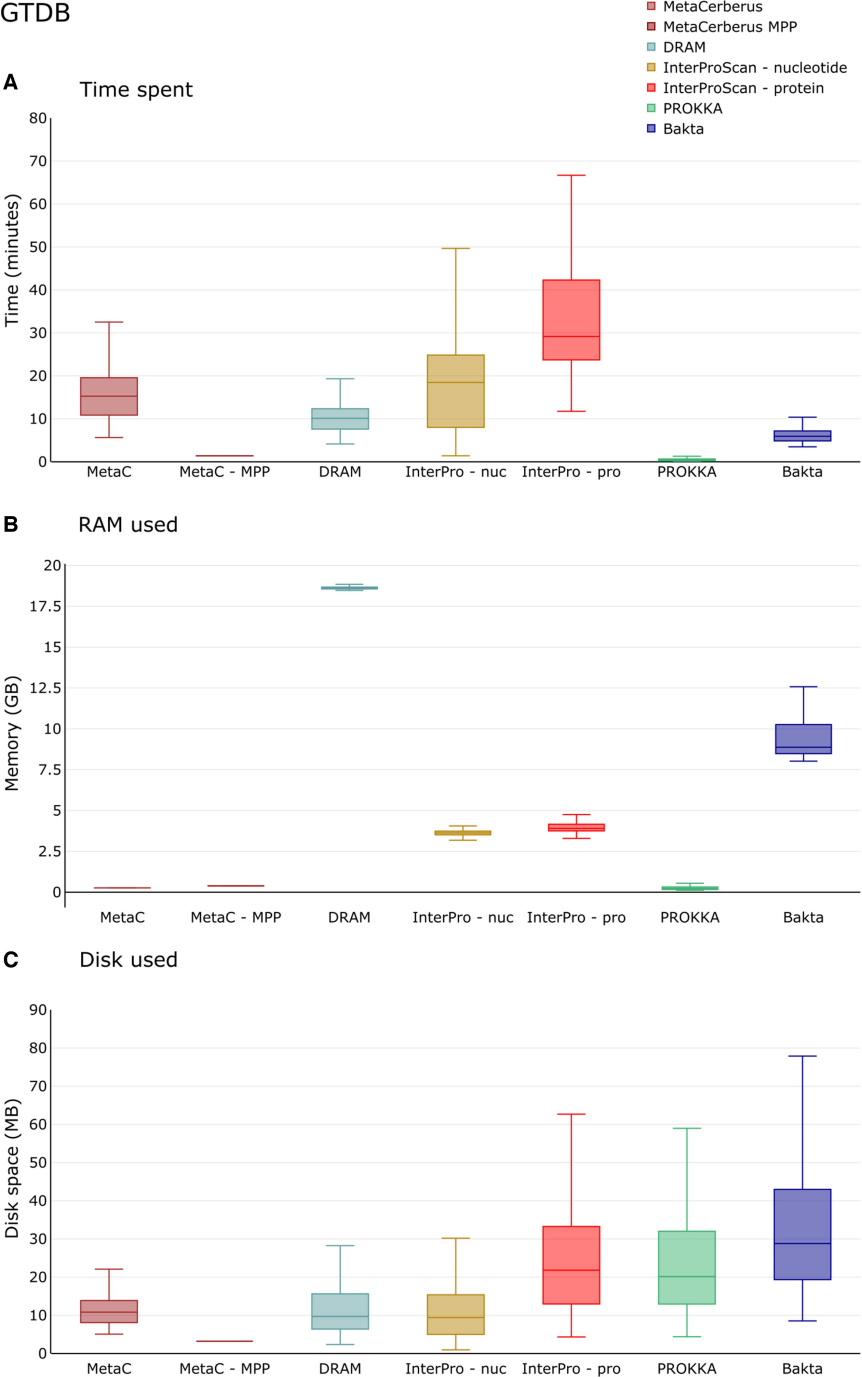
Computational resource comparison. DRAM, InterProScan, Prokka, and MetaCerberus are compared computationally for time to complete each genome annotation, RAM required to complete annotation per genome, and disc space needed for inputs/outputs. The 100 randomly selected bacterial and 100 archaeal genomes from GTDB (Supplementary Table S2) were the data. MPP testing for MetaCerberus utilized five nodes for comparisons with the group genomes option

We compared MetaCerberus to Bakta, DRAM, InterProScan, Pharokka, eggNOG-mapper, MicrobeAnnotator, and Prokka for the time used per genome, RAM utilization, and disk space used across 100 randomly selected bacterial and 100 archaea genomes within GTDB (Supplementary Table S2, Fig. 2). Generally, Prokka had the highest processing speed per genome (∼1.3 min median, Fig. 2). Our MetaCerberus massive parallel processing mode (MPP) was able to process a genome ∼1.3 min this is equivalent to Prokka speed (Fig. 2, Supplementary Table S3, *P* < 0.01). Bakta was faster than all other tools except Prokka and MetaCerberus MPP, at ∼5 min per genome (Fig. 2, Supplementary Table S3, *P* < 0.01). InterProScan either protein or nucleotide mode were the slowest at >18.5 min per genome median time (Fig. 2). DRAM was ∼5 min faster per genome than MetaCerberus without MPP (i.e. 10 min versus 15 min) (Fig. 2, Supplementary Table S3, *P* < 0.01). MicrobeAnnotator, InterproScan-nucleotide, InterproScan-protein, and eggNOG-mapper had the slowest average time when compared to the other tools (Fig. 2, Supplementary Fig. S5). MicrobeAnnotator was the slowest at median time of ∼503 min per genome; followed by InterproScan-protein at ∼29 min per genome (Fig. 2, Supplementary Fig. S5).

MetaCerberus and Prokka had the lowest RAM utilization at ∼0.2 GB; whereas other tools had higher RAM utilization (Fig. 2, Supplementary Table S3, *P* < 0.01). MicrobeAnnotator also had the highest RAM requirements at ∼88 GB per genome, which was 4.6-times higher than DRAM at 19 GB, and 293-times more than MetaCerberus (0.3 GB versus 88 GB) (Supplementary Fig. S5, Supplementary Table S3, *P* < 0.01). DRAM using default parameters had the second highest RAM observed (18.6 GB, followed by Bakta (∼9 GB), InterProScan (either mode, ∼3 to 3.5 GB), and eggNOG-mapper (∼1.5 GB) (Fig. 2, Supplementary Fig. S5, Supplementary Table S3, *P* < 0.01). MetaCerberus utilizes 5× less memory (0.3 GB versus 1.5 GB RAM) than the other HMM based tool eggNOG-mapper (Supplementary Fig. S5, Supplementary Table S3, *P* < 0.01). MetaCerberus also utilizes much less memory than DRAM (0.3 GB versus 19 GB), ∼63× less (Fig. 2, Supplementary Table S3, *P* < 0.01).

For disk space, MetaCerberus MPP provides the lowest amount at ∼3 MB of disk space per computation (Fig. 2, Supplementary Fig. S5). Bakta had the most disk space at ∼29 MB of disc followed by the other tools (Fig. 2, Supplementary Fig. S5). The standard mode of MetaCerberus provides more output files requiring more disc space overall when compared to MPP mode (Fig. 2, Supplementary Fig. S5).

### 3.3 Automatic statistical and pathway analysis

MetaCerberus provides automatic differential statistics, pathway gene enrichments, and KEGG map-based heatmaps in Pathview R for data exploration, data mining, and hypothesis generation. As a test for this functionality, we compared five distinct genospecies, *Rhizobium leguminosarum*, against five distinct *Exiguobacterium* spp. using MetaCereberus using both DESeq2 and edgeR (Supplementary Table S2). These genomes were selected as a comparison due to differences in metabolic function and phenotype to illustrate whether MetaCerberus could detect these differences or not. The *Rhizobium* selected have ability to fix nitrogen and can nodulate legume roots (i.e. *nod* genes) but lack carotenoid biosynthesis; whereas, *Exiguobacterium* lack nitrogen fixation genes and *nod* genes, but have the genes for carotenoid biosynthesis (64–66). MetaCerberus found differential pathway assignments using DESeq2 and pathview for carotenoid biosynthesis, ABC transporters, and phosphotransferase system (including nitrogen regulation) across the *Rhizobium* versus *Exiguobacterium* genome sets (Supplementary Figs S3, S6–S7). edgeR found an additional pathway in benzoate degradation that was not found in DESeq2 (Supplementary Fig. S8).

### 3.4 Annotation comparisons

Bakta, Pharokka, Prokka, DRAM, and MetaCerberus all use prodigal (or Pyrodigal) for pORF calling (67). MetaCerberus also provides an extra caller FragGeneScanRs and Prodigal-gv. InterProScan uses the emboss getorf pORF caller, which in all cases had lower pORFs than Prodigal regardless of the genome kingdom type (e.g. bacteria, archaea, CPR, phage, archaeal virus or eukaryotic virus) (Supplementary Fig. S9). Generally, Prokka, DRAM, and MetaCerberus had similar pORF calling numbers; however, DRAM did call more pORF from eukaryotic viruses (Supplementary Fig. S9).

**Figure 3.**
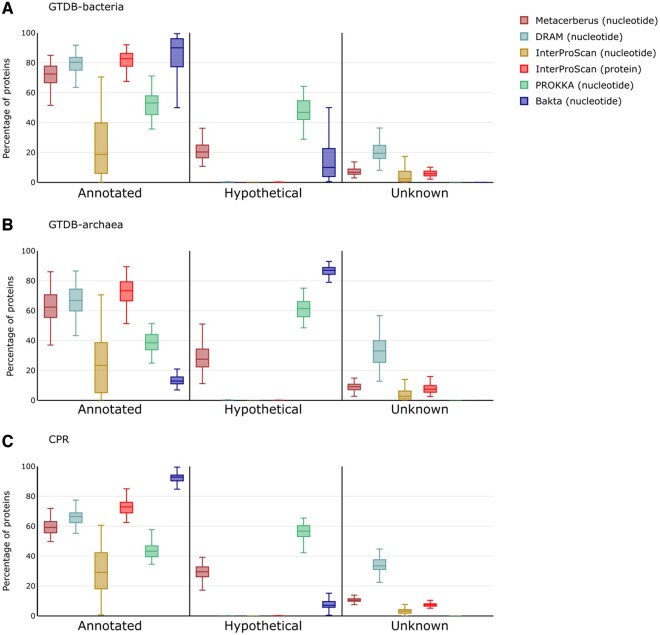
Annotation comparison across cellular domains of life (bacteria, archaea, CPR). MetaCerberus was compared to DRAM, InterProScan, and Prokka for annotation across various genomes. Supplementary Materials list The genomes for bacteria, archaea, and CPR (Supplementary Table S2)

**Figure 4.**
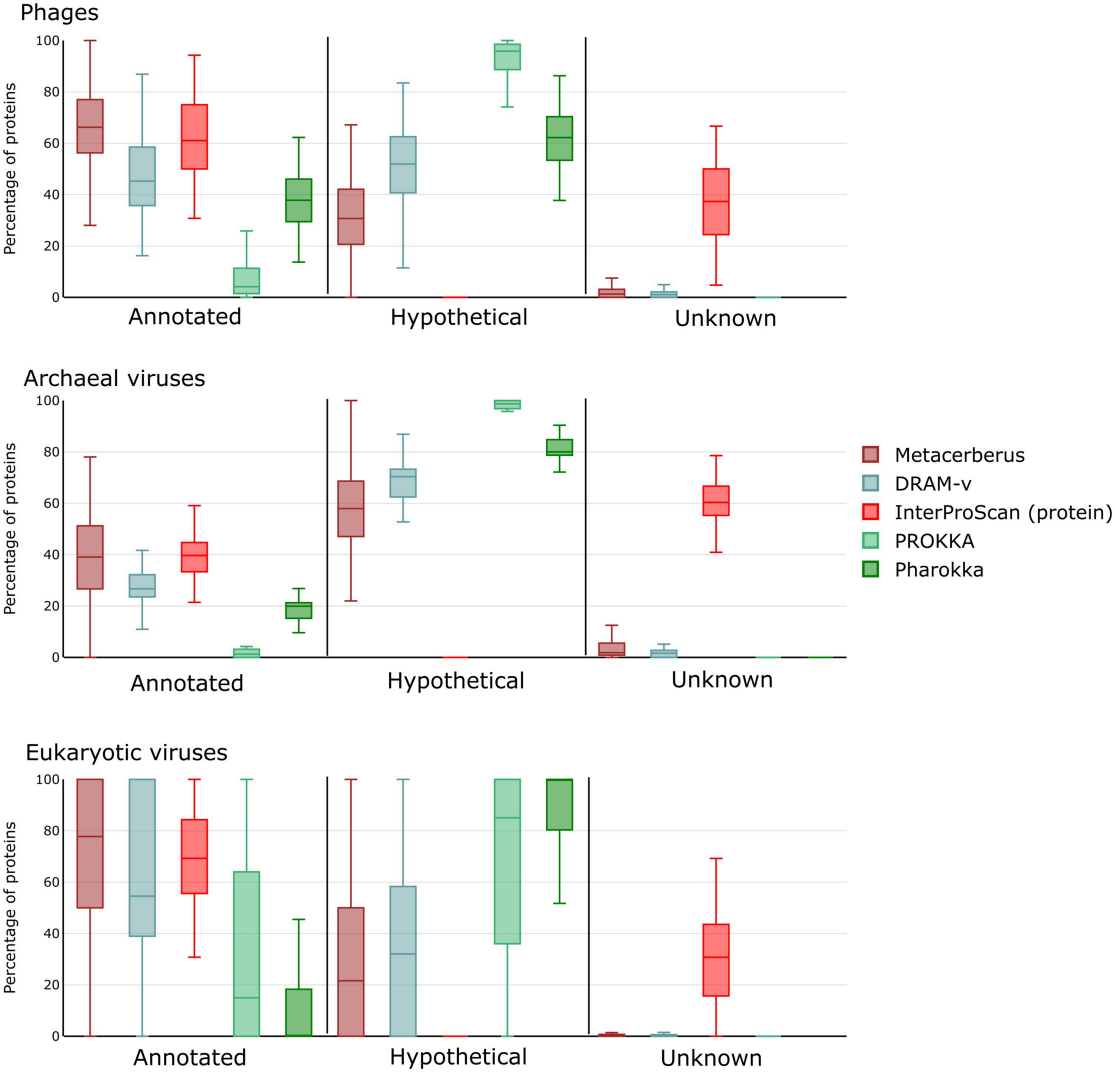
Annotation comparison across viral domains of life (phage, archaeal viruses, eukaryotic viruses). MetaCerberus was compared to DRAM-v, InterProScan, and Prokka for annotation across various genomes. The genomes are listed for phage, archaeal viruses, and eukaryotic viruses in Supplementary Materials (Supplementary Table S2)

**Figure 5.**
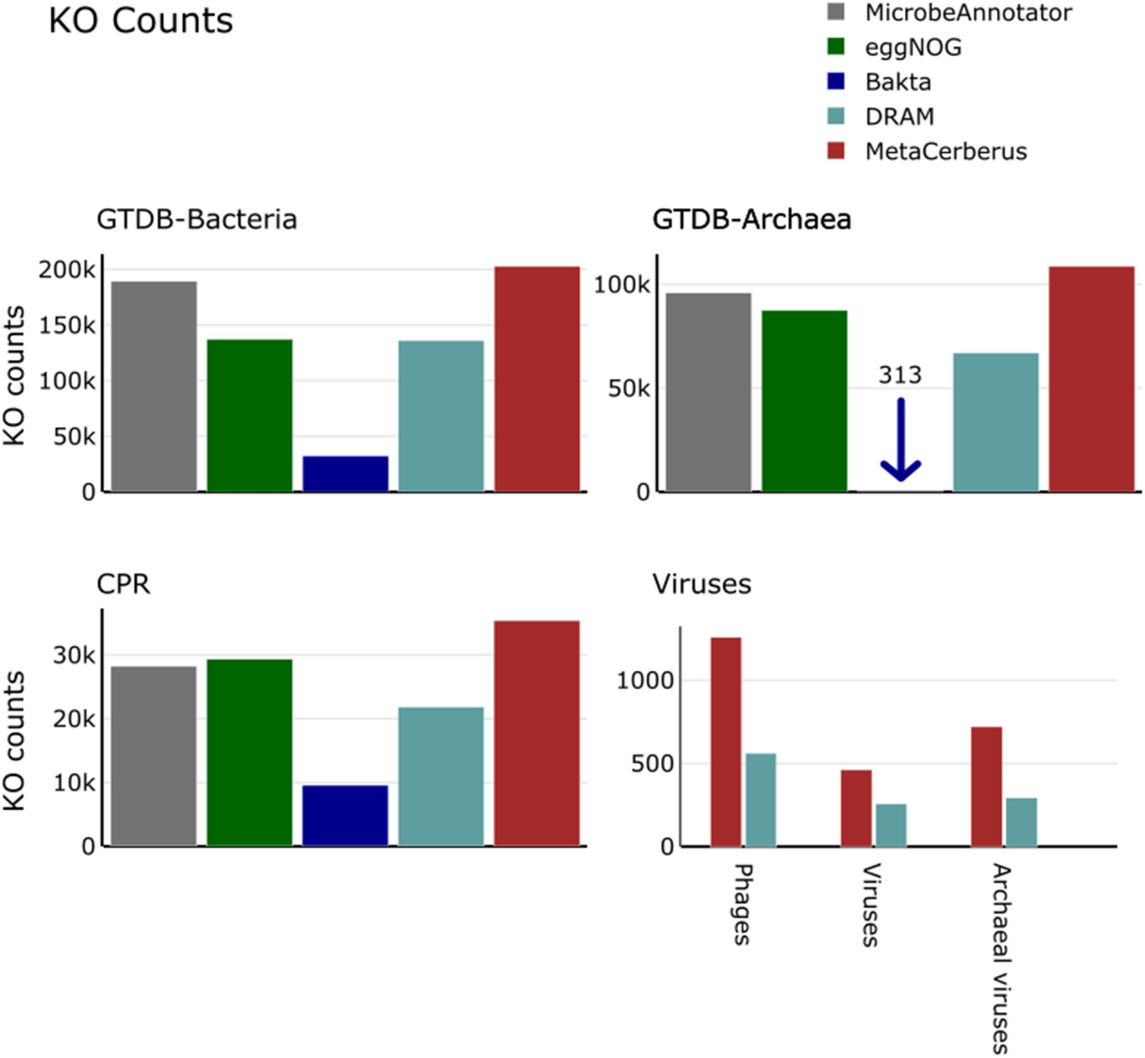
KEGG (KO) annotation comparison across tools. KO annotation comparison across tools and genomes from various domains of life. DRAM and MetaCerberus utilize KOfams for KEGG KO assignment if the user does not provide a KEGG KO database separately. The genomes for the comparison are listed in supplemental materials (Supplementary Table S2). The e-values and bitscore can vary between DRAM/DRAM-v and MetaCerberus. In this comparison, we choose the dbCAN e-value option of <1e^−15^ for DRAM/DRAM-v and MetaCerberus

Furthermore, we compared MetaCerberus to DRAM, InterProScan (in nucleotide and protein mode), Bakta, Pharokka, and Prokka for whether a pORF was annotated, listed as hypothetical, or unknown (no annotation). We randomly selected 100 unique bacterial and archaea genomes (i.e. 200 total) from GTDB, 100 unique phage genomes from INPHARED, 100 unique eukaryotic viral genomes from RefSeq, 78 CPR genomes, and 82 archaeal viral genomes for these annotation tests (Supplementary Table S2). For bacteria, Bakta had the highest median of annotation at >90% (Fig. 3). Bakta struggled with archaea annotation compared to MetaCerberus having the lowest media annotation at ∼13% with the highest hypothetical amounts ∼87% (Fig. 3, Supplementary Table S3, *P* < 0.01). MetaCerberus, DRAM, and InterProScan protein modes had similar annotation results of ∼73%–83% for bacteria, and 62%–73% with archaea (Fig. 3). InterProScan nucleotide had lower average annotations overall but had higher annotations for Archaea than Bakta, which found most hypotheticals for Archaea (Fig. 3). InterProScan using nucleotide mode had the lowest annotation amount across all kingdoms; this is due to the getorf caller for gene calling (Figs 3 and 4). Prokka had ∼50% of the pORFs as annotated and hypothetical for bacteria and ∼60% hypothetical for archaea (Fig. 3). CPR annotation Bakta had the highest at 90%, followed by InterProScan protein at ∼73%, then DRAM at 66%, then MetaCerberus at 59% (Fig. 3). MetaCerberus finds more KOs than DRAM, eggNOG-mapper, and Bakta for bacteria, archaea, and CPR (Fig. 5, Supplementary Table S3, *P* < 0.01). MetaCerberus, Bakta, and Prokka had fewer unknowns than DRAM for bacteria, archaea, and CPR genomes (Fig. 3). Prokka annotated very few CPR pORFs, with the majority >60% being hypothetical proteins (Fig. 3). DRAM generally does not find many hypothetical proteins or lists them as unknown.

MetaCerberus performs better for phages and archaeal viruses (Fig. 4). MetaCerberus annotates more per genome >66% phages and >39% archaeal viruses based on median value (Fig. 4, Supplementary Table S3, *P* < 0.01). Metacerberus and DRAM-v did not significantly differ on eukaryotic viral annotation, but differed significantly for phage and archaeal viruses (Fig. 4, Supplementary Table S3, *P* < 0.01). MetaCerberus outperforms viruses, phage and archaeal viruses annotation with more annotations, fewer hypotheticals, and fewer unknowns compared to Pharokka, InterProScan, and Prokka (Fig. 4, Supplementary Table S3, *P* < 0.01). Pharokka has difficulty with archaeal and eukaryotic viruses, it was designed for phages; however, MetaCerberus provides more annotations with less hypotheticals when directly compared (Fig. 4, Supplementary Table S3, *P* < 0.01). MetaCerberus annotates more KOs from KOfams than DRAM-v across archaeal viruses and phages (Fig. 5, Supplementary Table S3, *P* < 0.01). Pharokka, Prokka, and InterProScan do not provide KOs; therefore, we could not compare KOs found across domains to MetaCerberus. Bakta is designed for bacterial genomes, MAGs, and plasmids thus we did not compare against our viral datasets.

**Figure 6.**
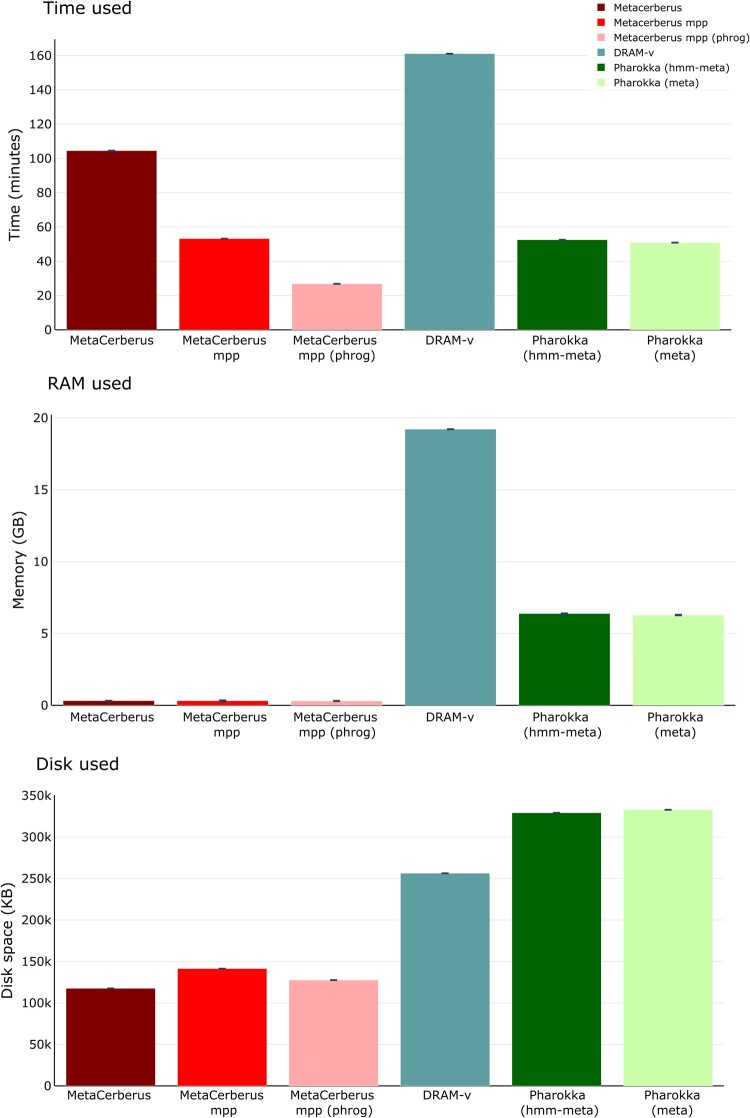
Virome computational resource comparison. We compared DRAM-v, Pharokka, MetaCerberus standard mode, MPP mode, and MPP with PHROG database only against a Swedish permafrost virome containing 1907 viral populations (VP). Tools were compared computationally for time to complete annotation, RAM required to complete annotation, and disc space needed for inputs/outputs. MPP testing for MetaCerberus utilized five nodes for comparisons

We compared MetaCerberus against DRAM-v and Pharokka, which are tools that specialize for viruses and phages. A virome containing 1907 viral populations (VPs) obtained from Swedish permafrost used in the DRAM paper was utilized for this comparison. Based on time, MetaCerberus standard took ∼105 min to complete the annotation compared to ∼160 min for DRAM-v (Fig. 6, Supplementary Table S3, *P* < 0.05). When MetaCerberus is utilized at its full potential with RAY it only takes ∼53 min for the same dataset (Fig. 6). Pharokka is faster than MetaCerberus standard (∼105 min versus ∼53 min) (Fig. 6, Supplementary Table S3, *P* < 0.05). Pharokka (1.5 version) using hmm-meta or meta options have similar speeds to MetaCerberus MPP (∼53–50 min), but only annotate PHROGs, whereas MetaCerberus does offer annotations for many more databases in MPP (e.g. CAZy/dbCAN, KEGG/KO, VOG/pVOG, COG) (Fig. 6, Supplementary Table S3, *P* < 0.05). If we run PHROG database only with MetaCerberus MPP, the time is ∼50% faster than Pharokka at ∼27 min (Fig. 6, Supplementary Table S3, *P* < 0.05).

RAM utilization was much less with MetaCerberus with or without MPP than DRAM-v, both in MPP and non-MPP mode, with 0.3 GB of RAM compared to 19 GB with DRAM-v, and ∼6 GB with Pharokka (Fig. 6, Supplementary Table S3, *P* < 0.05). MetaCerberus had more annotations than DRAM-v for the Swedish permafrost virome across shared databases (i.e. KO, CAZy, and VOG) (Supplementary Fig. S10).

## 4 Discussion

MetaCerberus provides a generalizable low memory, robust, scalable, and rapid annotation across the tree of life, exclusively using HMMs/HMMER. HMMER is a powerful tool to find pORFs that may be missed by standard homology-based tools due to its protein-based domain centricity and supervised machine-learning nature. It's rarely used elusively in many tools due to the speed and time required to finish annotation. MetaCerberus has provided a solution to this scaling issue using RAY and items needed from our kmer counter MerCat2. eggNOG-mapper v2 is the only tool that exclusively provides HMMs/HMMER-based for across the tree of life (i.e. bacteria to virus) annotation. MetaCerberus runs 1.3× as fast on a single node than eggNOG-mapper v2 without RAY, on 7.5× less memory. MetaCerberus MPP with RAY is 15× as fast as eggNOG-mapper v2 on a one-third-size database.

MetaCerberus detected differences in metabolic function between two sets of genomes: *Rhizobium* versus *Exiguobacterium* using functional gene analysis and pathway enrichment. Metabolically these bacteria are differential including *Rhizobium* with nitrogenase and *nod* genes, but without carotenoid biosynthesis; whereas, *Exiguobacterium* have carotenoid biosynthesis but lack nitrogenase and *nod* genes (White III *et al.* 2018, Jinendiran *et al.* 2020, Young *et al.* 2021). MetaCerberus also detected that the genome sets were differential for benzoate degradation, which would have not been found otherwise, highlighting MetaCerberus as a tool for investigative hypothesis generation.

Generally, MetaCerberus performs better for archaeal viruses and phage annotation when directly compared to DRAM-v and Pharokka. DRAM-v finds more pORFs than MetaCerberus (Supplementary Fig. S9) due to it using the -meta option in Prodigal; whereas, MetaCerberus but still can annotate archaeal viruses and phage genomes better than DRAM-v on a much smaller database (Supplementary Fig. S10). Viruses, archaeal viruses, and phages are a grand challenge to unlock the ‘unknown’ and ‘hypothetical’ functions within their genomes.

As data scales, computational time, memory, and waiting for results will take longer. Scalable tools like MetaCerberus are needed as we approach Petabyte levels of sequencing. MetaCerberus provides a further community resource to annotate the unknowns of our biosphere. Lastly, MetaCerberus provides a robust tool kit to annotate the entire tree of life at scale.

Contributing to MetaCerberus and Fungene: MetaCerberus as a community resource as recently acquired Fungene (Fish *et al.* 2013), we welcome contributions of other experts expanding annotation of all domains of life (viruses, bacteria, archaea, eukaryotes). Please send us an issue on our MetaCerberus GitHub (www.github.com/raw-lab/metacerberus/issue); we will fully annotate your genome, add suggested pathways/metabolisms of interest, make custom HMMs to be added to MetaCerberus and FunGene.

## Supplementary Material

btae119_Supplementary_Data

## Data Availability

Sequence files, genome files, and supplemental data are available at https://osf.io/3uz2j/. Databases are also freely available at https://osf.io/3uz2j/. All code is available at www.github.com/raw-lab/metacerberus.
