# Supplementary figures and images for: MetaCerberus: distributed highly parallelized HMM-based processing for robust functional annotation across the tree of life
Source: Bioinformatics. 2024 Feb 29;40(3):btae119. doi: 10.1093/bioinformatics/btae119 (PMC10955254; doi:10.1093/bioinformatics/btae119)

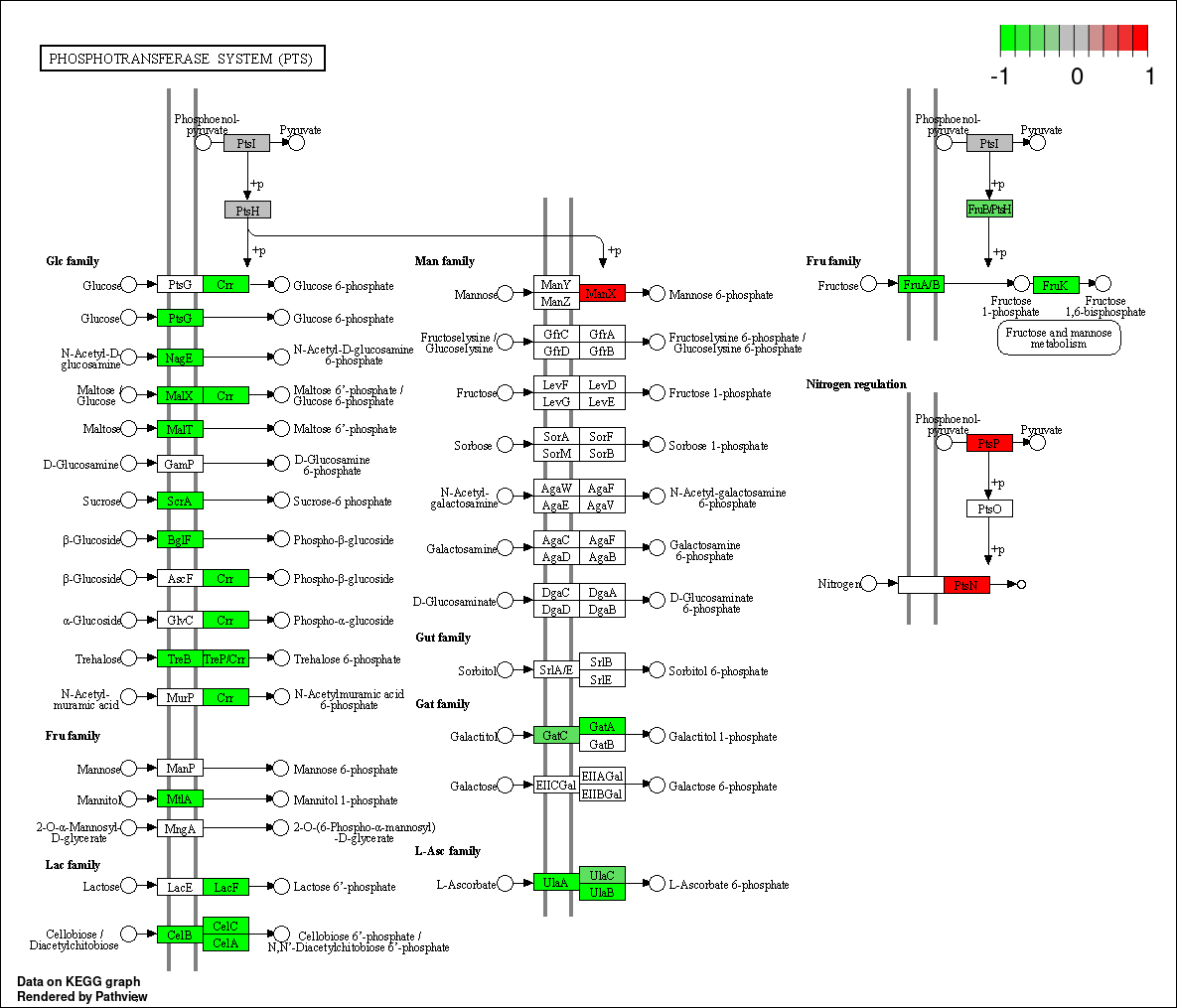

Supplement: btae119_Supplementary_Data [file btae119_supplementary_data.zip › Fig-S7.png]

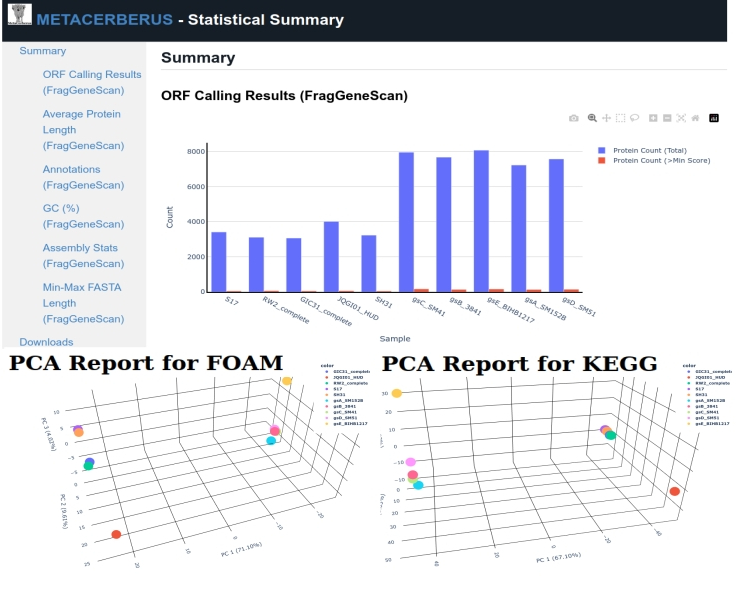

Supplement: btae119_Supplementary_Data [file btae119_supplementary_data.zip › Fig-S4.jpg]

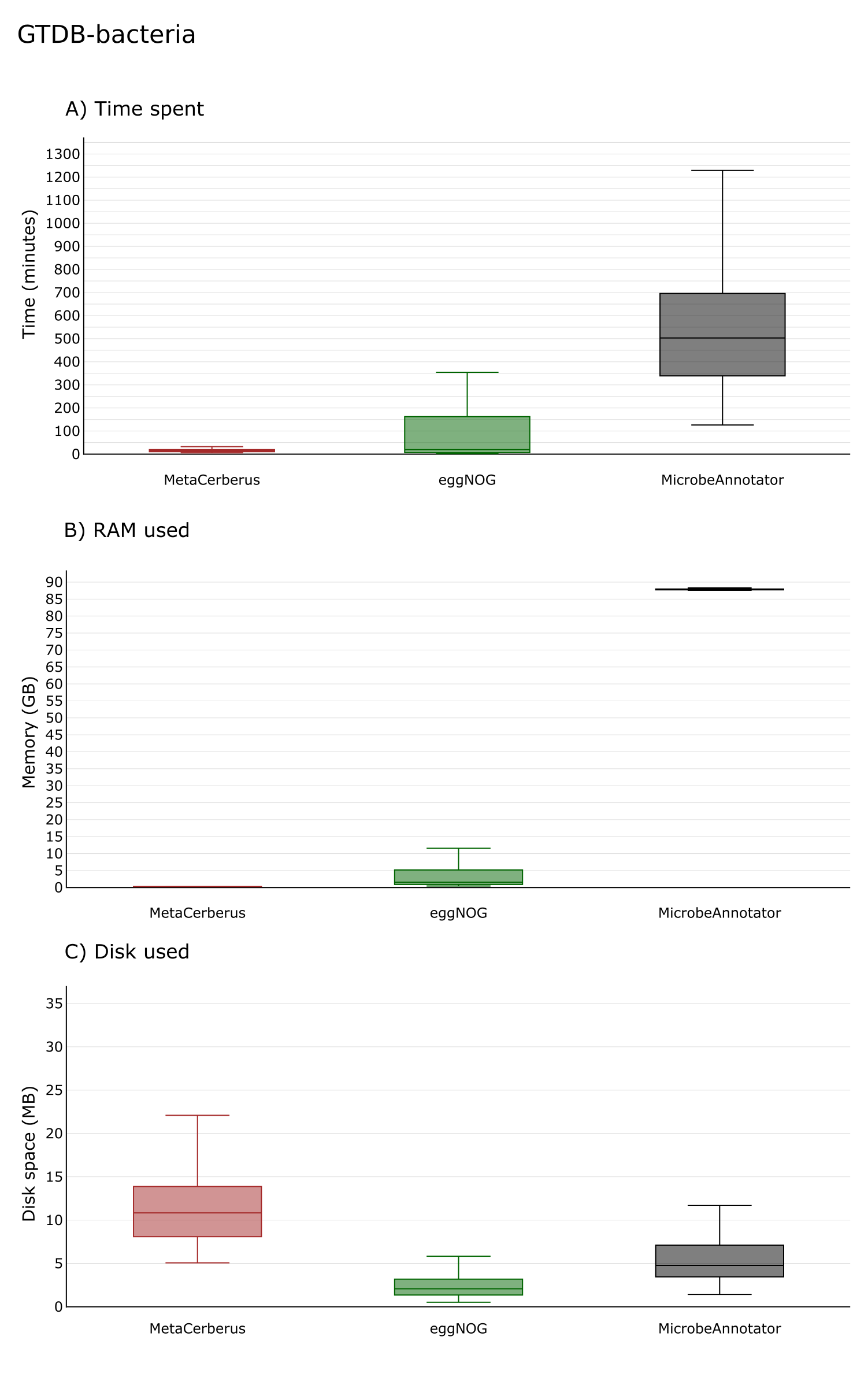

Supplement: btae119_Supplementary_Data [file btae119_supplementary_data.zip › Fig-S5.png]

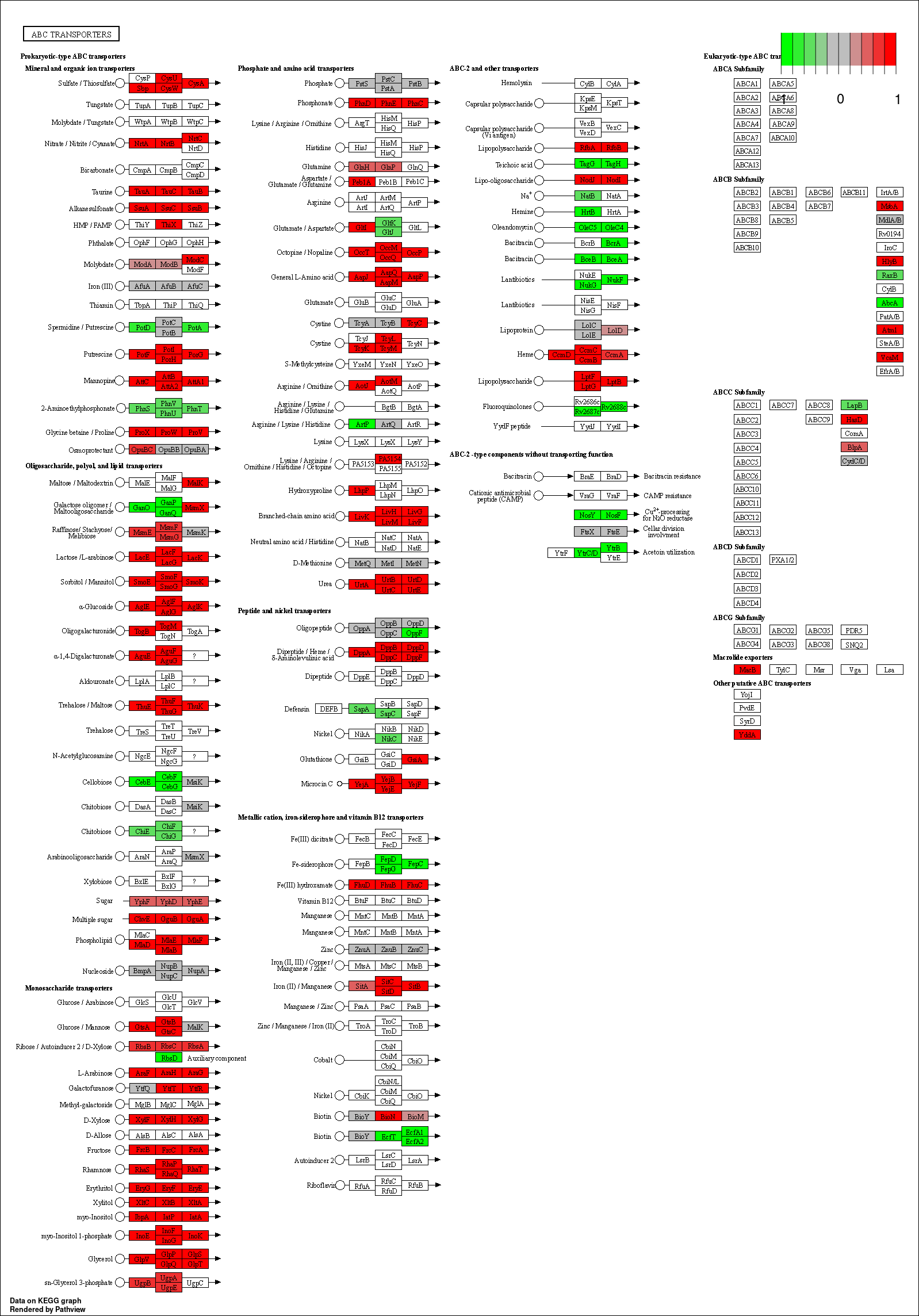

Supplement: btae119_Supplementary_Data [file btae119_supplementary_data.zip › Fig-S6.png]

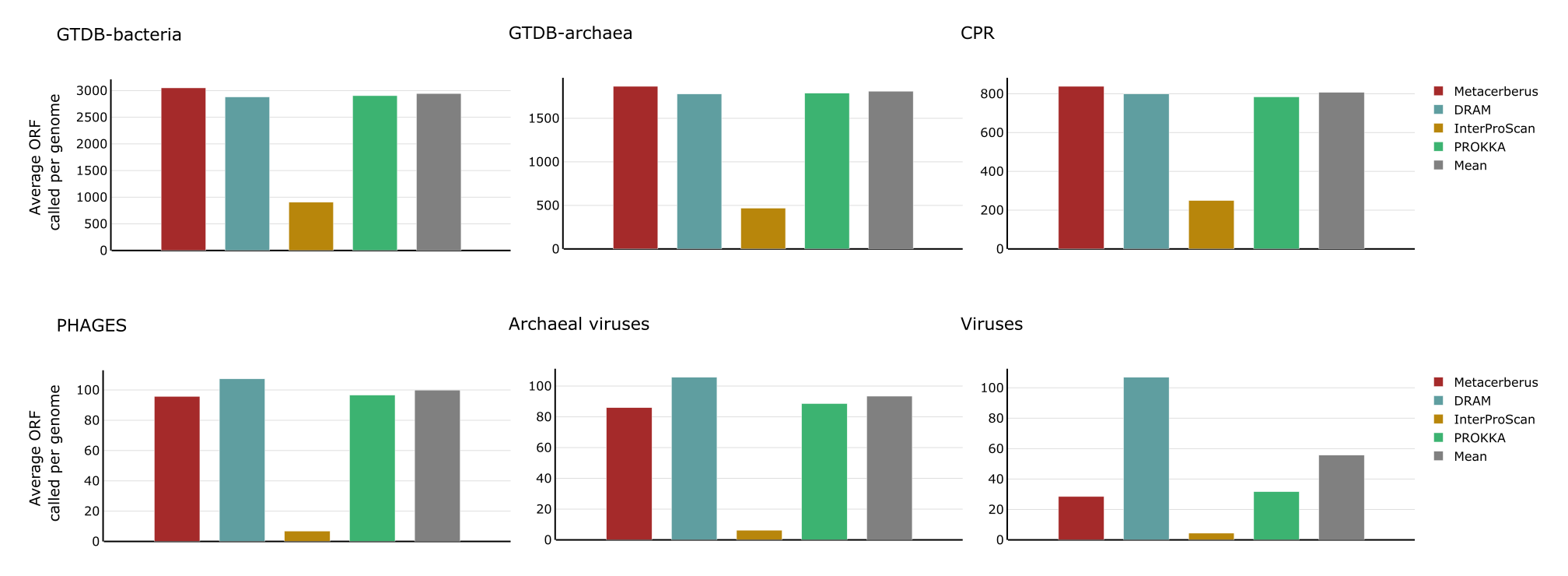

Supplement: btae119_Supplementary_Data [file btae119_supplementary_data.zip › Fig-S9.png]

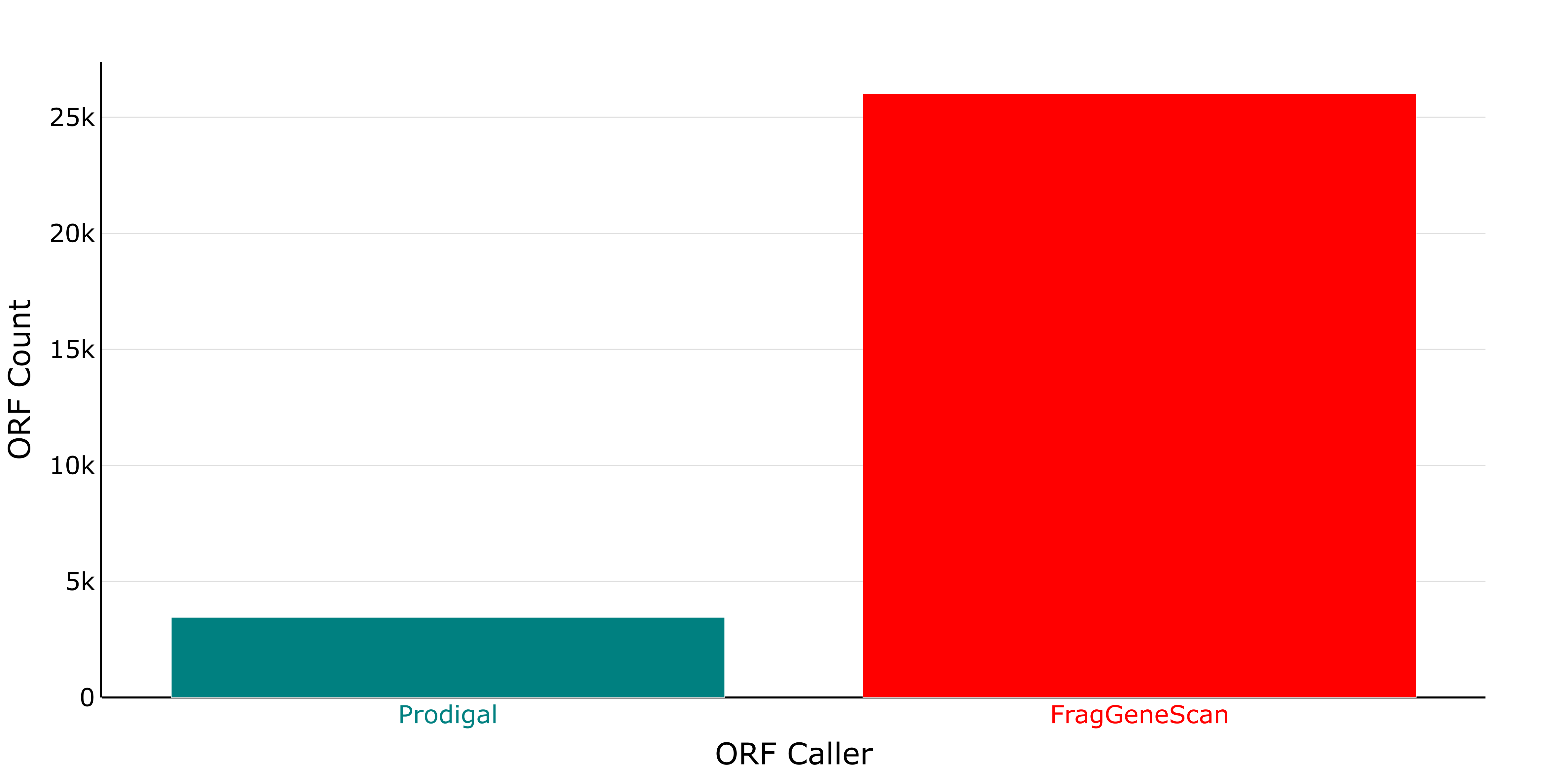

Supplement: btae119_Supplementary_Data [file btae119_supplementary_data.zip › Fig-S1.png]

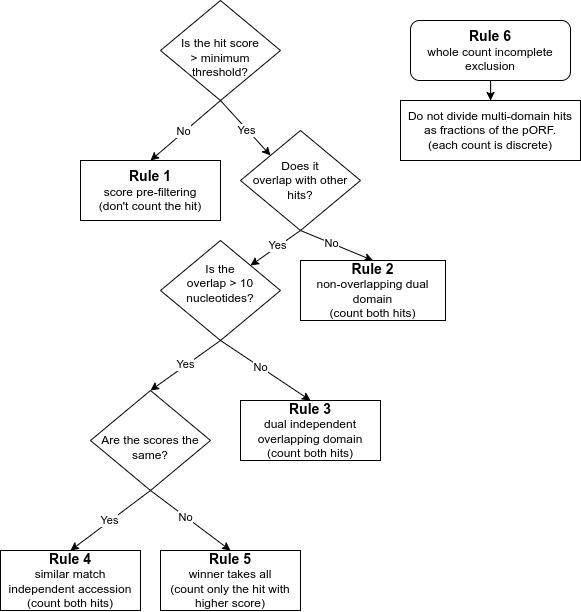

Supplement: btae119_Supplementary_Data [file btae119_supplementary_data.zip › Fig-S2.jpg]

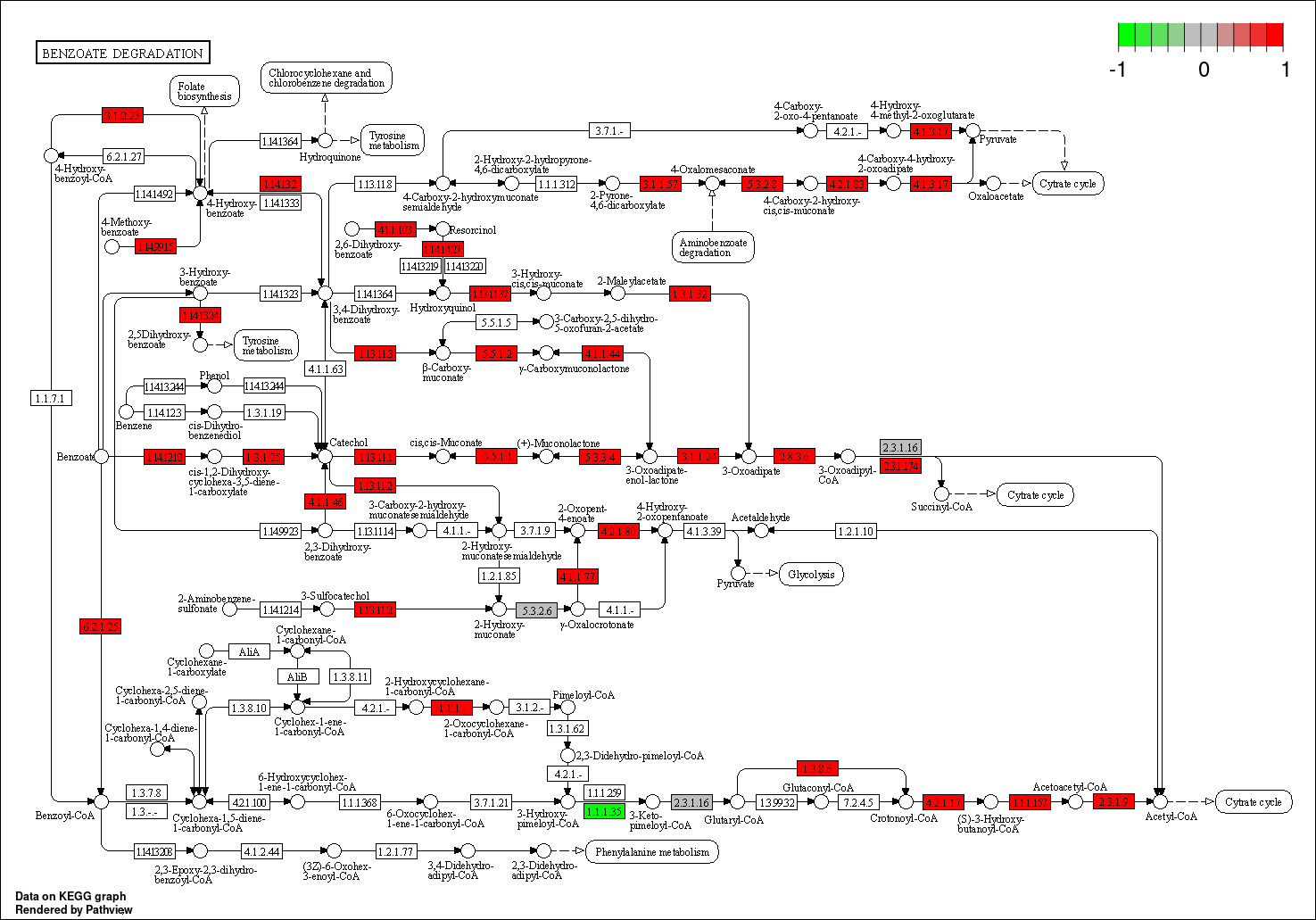

Supplement: btae119_Supplementary_Data [file btae119_supplementary_data.zip › Fig-S8.png]

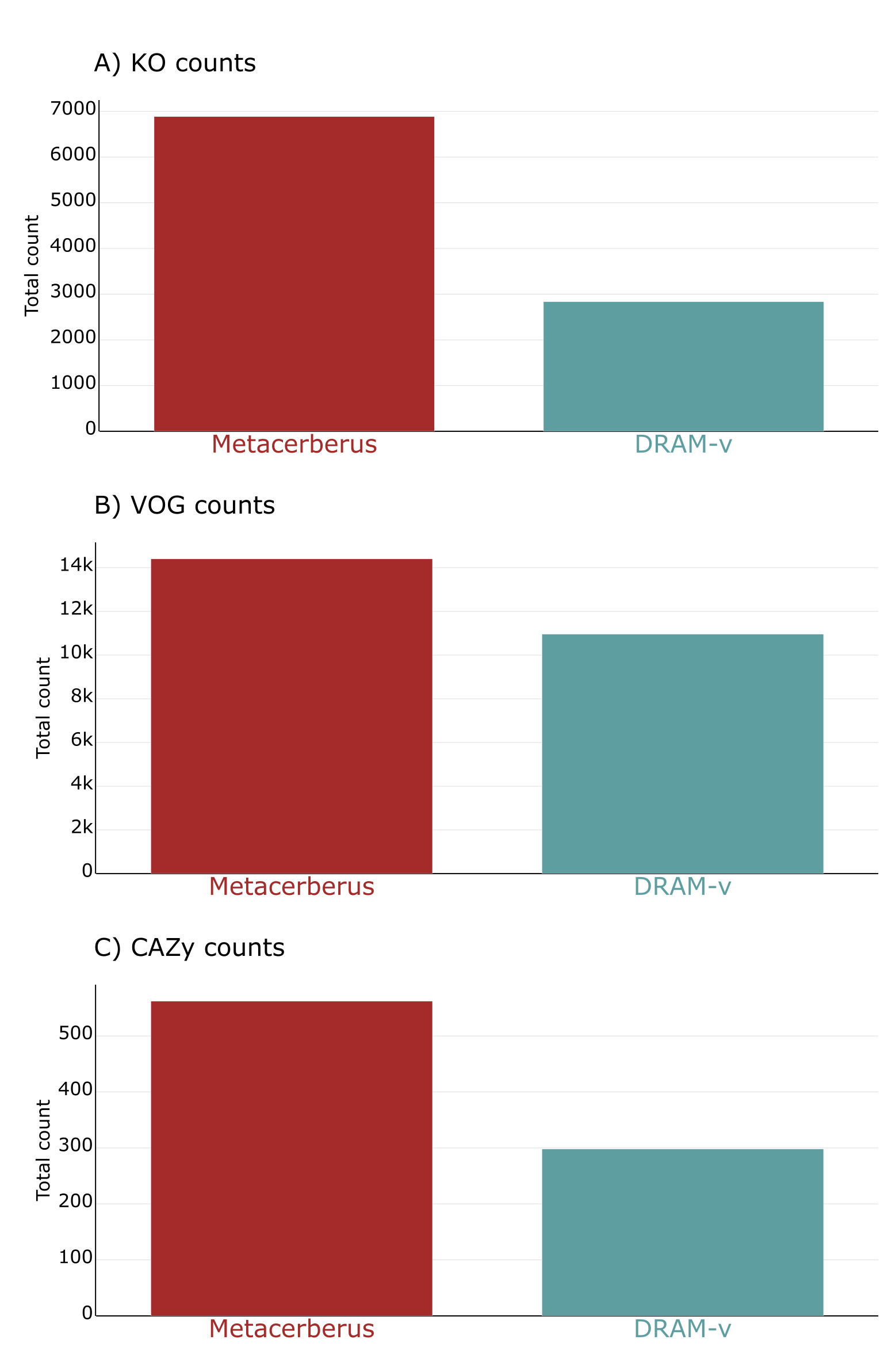

Supplement: btae119_Supplementary_Data [file btae119_supplementary_data.zip › FigureS10.png]

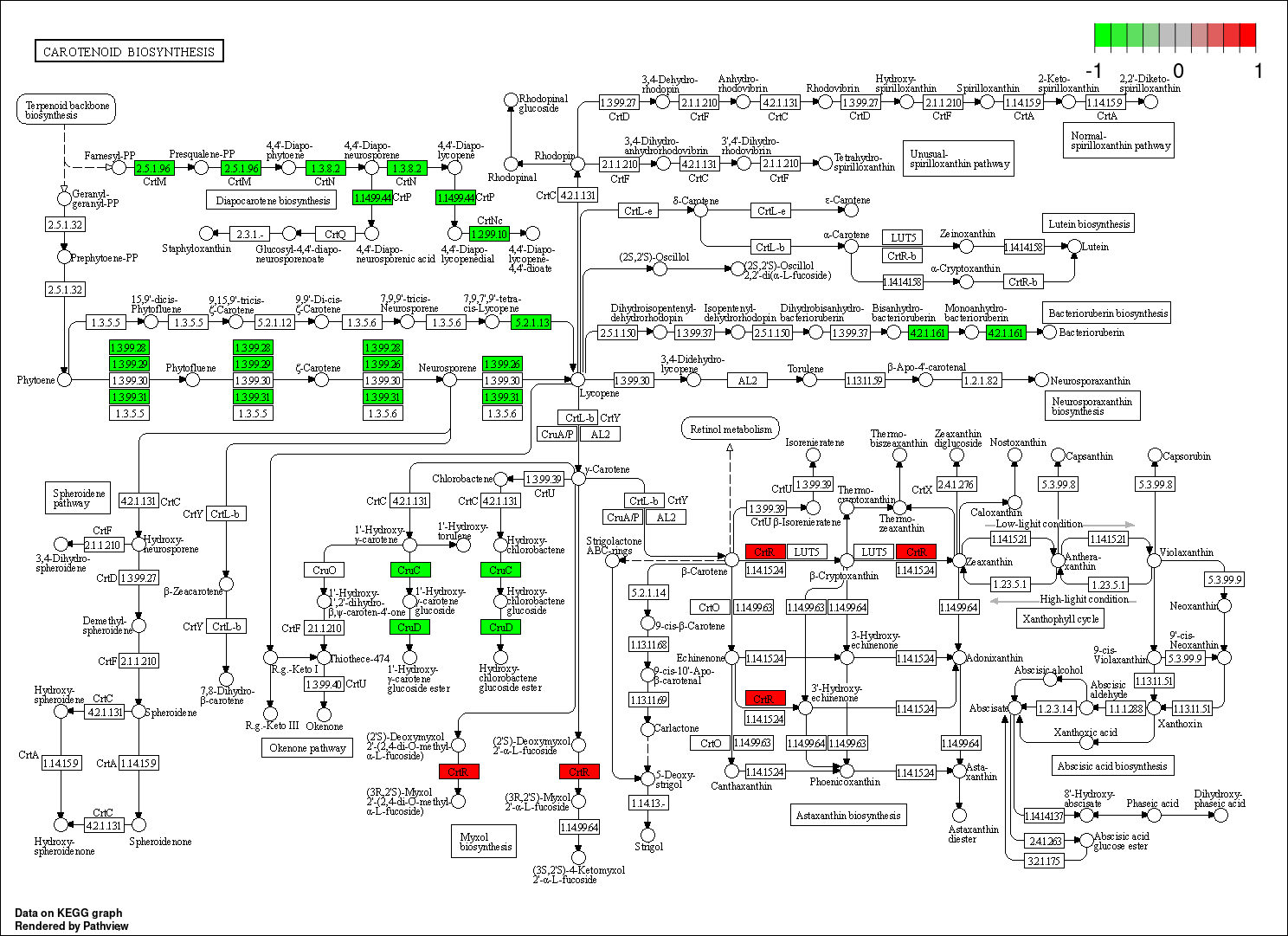

Supplement: btae119_Supplementary_Data [file btae119_supplementary_data.zip › Fig-S3.png]
